# *In situ* generation of dendritic cell vaccines within 3D-printed scaffolds

**DOI:** 10.1093/nsr/nwag127

**Published:** 2026-03-03

**Authors:** Liming Bian

**Affiliations:** School of Biomedical Sciences and Engineering, Guangzhou International Campus, South China University of Technology, China

**Keywords:** dendritic cell vaccines, 3D-printed scaffolds, *in situ* generation

Dendritic cell (DC) vaccines have long been hailed as a promising strategy for cancer immunotherapy due to their unique ability to present tumor antigens and prime naive T cells [1]. However, the clinical translation of this technology has been stymied by a fundamental logistical hurdle: the reliance on complex *ex vivo* manufacturing [2]. The traditional paradigm—isolating monocytes, differentiating them into DCs, and loading them with antigens in a cleanroom facility—is labor-intensive, costly and difficult to standardize. In a recent study published in *National Science Review*, Lang Rao, Qi Li and colleagues present a paradigm-shifting solution: an *in situ*-generated DC vaccine (isDCV) that transfers the entire cell culture and education process from the Petri dish directly to the *in vivo* environment [3].

The core innovation of this work lies in its precise orchestration of cell fate decisions within a synthetic niche. The authors prepared a gelatin methacryloyl (GelMA) hydrogel with a distinct porous structure (Fig. 1A) and utilized 3D printing technology to fabricate a customized scaffold (Fig. 1B). Scanning electron microscopy (SEM) analysis confirmed that this 3D-printed hydrogel possesses a highly interconnected architecture with excellent cell-loading capacity (Fig. 1C). This structural design allows the scaffold to effectively co-deliver undifferentiated bone marrow mononuclear cells (BM-MNCs) alongside a specific cocktail of regulatory cues, serving not merely as a delivery vehicle, but as an *in vivo* bioreactor that spatially and temporally controls two critical biological processes: differentiation and maturation.

**Figure 1.**
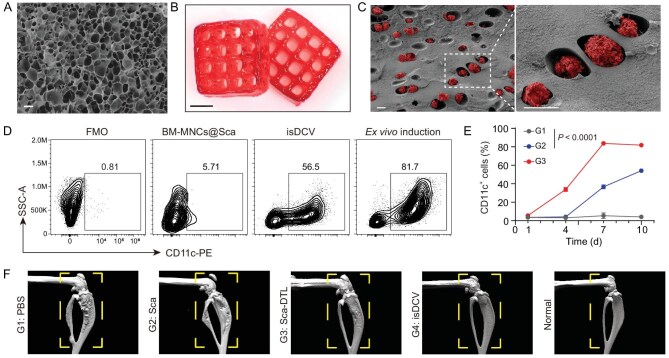
(A) SEM image of GelMA hydrogel. Scale bar, 10 μm. (B) The manufacturing and stability of the 3D porous scaffold. Scale bar, 5 mm. (C) SEM image of isDCV. Scale bars, 10 μm. (D) Representative flow cytometry analysis of DCs in live cells in BM-MNCs@Sca, isDCV and culture dish (*ex vivo* induction) on Day 10, with fluorescence minus one (FMO). (E) Change of quantitation of the percentage of DCs in BM-MNCs@Sca (G1), isDCV (G2) and *ex vivo* induction (G3) over time. (F) Representative micro-CT images of the femurs and tibias in each group. All data are expressed as mean ± SD (*n* = 4). Statistical significance was calculated via ordinary one-way analysis of variance (ANOVA) with a Tukey’s test. Reproduced from Chen *et al.* [3] with permission.

First, the scaffold establishes a bioactive 3D microenvironment enriched with granulocyte-macrophage colony-stimulating factor (GM-CSF) and Flt-3 ligand (FLT3L), which effectively drives the *in situ* differentiation of encapsulated BM-MNCs into the DC lineage over 7–10 days (Fig. 1D and E). While the unique porous architecture supports cell viability during this gradual phenotypic transition, the system simultaneously ensures functional maturation by incorporating X-ray-irradiated tumor lysates (DTLs) into the bioink. By exposing the nascent cells to damage-associated molecular patterns (DAMPs), the scaffold triggers the upregulation of co-stimulatory molecules (CD80, CD86) and MHC-II, thereby transforming the newly differentiated cells into active antigen-presenting cells capable of robust lymph node homing.

By seamlessly coupling *in situ* differentiation with DAMP-mediated maturation, the isDCV strategy effectively bypasses the fragility and variability of *ex vivo* handling. The result is a robust antitumor immune response that not only clears residual tumor cells at the surgical site but also establishes systemic memory. Notably, in murine models, the isDCV successfully inhibited osteoblastic lesions caused by prostate cancer bone metastasis (Fig. 1F), proving its efficacy against complex metastatic conditions. This work elegantly demonstrates that with the right biomaterial design, the host organism can be empowered to manufacture its own living drugs, marking a significant step forward for the development of personalized cancer immunotherapies.

## References

[1] Liu YJ, Kanzler H, Soumelis V et al. Nat Immunol 2001; 2: 585–9. 10.1038/89726 11429541

[2] Sabado RL, Balan S, Bhardwaj N. Cell Res 2017; 27: 74–95. 10.1038/cr.2016.157 28025976 PMC5223236

[3] Chen L, Xu Y, Hu X et al. Natl Sci Rev 2026; 13: nwag037. 10.1093/nsr/nwag037 41710125 PMC12912718

